# Milnacipran inhibits glutamatergic *N*-Methyl-D-Aspartate receptor activity in Spinal Dorsal Horn Neurons

**DOI:** 10.1186/1744-8069-8-45

**Published:** 2012-06-19

**Authors:** Tatsuro Kohno, Masafumi Kimura, Mika Sasaki, Hideaki Obata, Fumimasa Amaya, Shigeru Saito

**Affiliations:** 1 Department of Anesthesiology, Niigata University Graduate School of Medical and Dental Sciences, 757 Asahimachi, Chuo ku Niigata, 951-8510, Japan; 2 Department of Anesthesiology, Gunma University Graduate School of Medicine; 3 Department of Anesthesiology, Kyoto Prefectural University of Medicine, 757 Asahimachi, Chuo ku Niigata, 951-8510, Japan; 4 Pain Mechanism Research Group, 757 Asahimachi, Chuo ku Niigata, 951-8510, Japan

**Keywords:** Antidepressants, *N*-methyl-D-aspartate (NMDA) Receptor, Spinal Analgesia

## Abstract

**Background:**

Antidepressants, which are widely used for treatment of chronic pain, are thought to have antinociceptive effects by blockade of serotonin and noradrenaline reuptake. However, these drugs also interact with various receptors such as excitatory glutamatergic receptors. Thermal hyperalgesia was induced by intrathecal injection of NMDA in rats. Paw withdrawal latency was measured after intrathecal injection of antidepressants. The effects of antidepressants on the NMDA and AMPA-induced responses were examined in lamina II neurons of rat spinal cord slices using the whole-cell patch-clamp technique. The effects of milnacipran followed by application of NMDA on pERK activation were also investigated in the spinal cord.

**Results:**

Intrathecal injection of milnacipran (0.1 μmol), but not citalopram (0.1 μmol) and desipramine (0.1 μmol), followed by intrathecal injection of NMDA (1 μg) suppressed thermal hyperalgesia. Milnacipran (100 μM) reduced the amplitude of NMDA (56 ± 3 %, 64 ± 5 % of control)-, but not AMPA (98 ± 5 %, 97 ± 5 % of control)-mediated currents induced by exogenous application and dorsal root stimulation, respectively. Citalopram (100 μM) and desipramine (30 μM) had no effect on the amplitude of exogenous NMDA-induced currents. The number of pERK-positive neurons in the group treated with milnacipran (100 μM), but not citalopram (100 μM) or desipramine (30 μM), followed by NMDA (100 μM) was significantly lower compared with the NMDA-alone group.

**Conclusions:**

The antinociceptive effect of milnacipran may be dependent on the drug’s direct modulation of NMDA receptors in the superficial dorsal horn. Furthermore, in addition to inhibiting the reuptake of monoamines, glutamate NMDA receptors are also important for analgesia induced by milnacipran.

## Background

It is well established that antidepressants have antinociceptive effects; because of this, they are widely used for treatment of chronic pain [1]. In particular, tricyclic antidepressants (TCAs) have long been the mainstay of treatment for neuropathic pain, which is due to lesion or dysfunction of the peripheral or central nervous system. Antidepressants have the unique ability to inhibit the presynaptic reuptake of monoamines, serotonin (5-HT), and noradrenaline (NA) at the neuronal terminals [2], and this activity can produce antinociceptive effects. Recently, more selective monoamine reuptake inhibitors, such as 5-HT and NA reuptake inhibitors (SNRIs) and selective 5-HT reuptake inhibitors (SSRIs), have been introduced and are clinically used to treat neuropathic pain [1]. However, the underlying mechanisms of these drugs may be more complex than simply the blockade of 5-HT and NA reuptake.

In fact, TCAs could also interact with various receptors including *N*-methyl-D-aspartate (NMDA) receptors to produce nociceptive effects. NMDA glutamate receptors are one of the major receptor channel types mediating rapid excitatory neurotransmission in the central nervous system, and they also play an important role in central sensitization regarding long-term pain [3,4]. Some previous studies have suggested that there are interactions in nociceptive transmission between TCAs and NMDA receptors in the spinal cord [5]. However, there have been no studies demonstrating that the antinociceptive effects of antidepressants are attributable to the inhibition of NMDA receptors in the spinal cord at the cellular level.

The spinal cord is an important site of action of antidepressant-mediated antinociception [6]. The brainstem-spinal descending 5-HT and NA systems suppress nociceptive signals from primary afferent neurons to the spinal dorsal horn. Concurrently, the superficial dorsal horn preferentially receives nociceptive primary afferent fibers. Thus, the spinal dorsal horn is thought to play an important role in modulating nociceptive transmission from the periphery [7,8] as well as in regulating the antinociceptive effect of antidepressants.

The purpose of the current study was to test the hypothesis that 3 different antidepressants, milnacipran as a SNRI, citalopram as a SSRI, and desipramine as a TCA (a preferential noradrenergic reuptake inhibitor) have a direct antagonistic effect on NMDA and AMPA-mediated responses in the spinal dorsal horn.

## Methods

### **Surgical Preparation**

The study was approved by the Animal Care and Use Committee of the Gunma University School of Medicine (Maebashi, Japan). Male rats (250–270 g) were used in all experiments. Animals were housed under a 12-h light–dark cycle with food and water ad libitum. For intrathecal administration, a sterilized 32-gauge polyethylene catheter (ReCathCo, Allison Park, PA) connected to an 8.5-cm Tygon external tubing (Saint-Gobain Performance Plastics, Akron, OH) was inserted under isoflurane anesthesia, as previously described [9]. The catheter was passed caudally 8.0 cm from the cisterna magnum to the lumbar enlargement. The animals were allowed to recover for 1 week before being used experimentally. Only animals without evidence of neurologic dysfunction after catheter insertion were used for all studies.

### **Testing Procedures**

The thermal nociceptive threshold was measured with a device (Plantar Test®, IITC Inc. Life Science, Woodland Hills, CA) using a method similar to that reported previously [10]. Rats were placed in individual plastic boxes (10 × 20 × 24 cm) on the glass surface of the testing apparatus, which was maintained at 30 °C during all testing, and were allowed to acclimate for 30 min. Paw withdrawal latency (PWL) was determined using an intense light focused on the hind paw, as previously described [10]. Light intensity was adjusted so that baseline latency was between 9 and 11 s in all animals. A cutoff of 20 s was selected to avoid tissue damage during periods of analgesia, but no animals reached this cutoff point.

The thermal hyperalgesic state was induced by intrathecal injection of 1 μg NMDA. The dose was selected according to a previous study [11]. This experiment is most specific to evaluate the effect of antidepressants on NMDA-mediated responses in the spinal cord. PWL was measured three times in the right or left foot in the middle of the footpad. These three observations were averaged for each animal. PWLs were measured before and after intrathecal injection of milnacipran, citalopram, desipramine, or saline (six animals in each group), and NMDA was injected intrathecally 15 min after the injection (time 0). PWLs were measured at 0, 30, 60, 90, and 120 min after intrathecal injection of NMDA.

### **Drugs and Their Administration**

The agents administered in this study were milnacipran, citalopram, desipramine, and NMDA. Each antidepressant or saline was administered intrathecally 15 min prior to NMDA injection. Drugs were administered intrathecally in a volume of 5 μl, followed by an injection of 10 μl of saline to flush the catheter. All drugs were dissolved in normal saline. The doses of milnacipran were selected according to the previous studies [12,13]. The maximum dose of milnacipran (0.1 μmol) was used for desipramine and citalopram injections because the doses to produce analgesia in these 3 drugs are almost same [14,15].

### ***In Vitro*Patch-Clamp Recordings**

This portion of the study was approved by the Animal Care and Use Committee at Niigata University Graduate School of Medical and Dental Sciences (Niigata, Japan). Male rats (150–200 g) were anesthetized with urethane (1.5 g/kg, i.p.). A dorsal laminectomy was performed, and the lumbosacral segment of the spinal cord with ventral and dorsal roots attached was removed [16,17]. The rats were then immediately killed by exsanguination, and the spinal cords were placed in pre-oxygenated ice-cold Krebs solution. After the arachnoid membrane was removed, the spinal cord was placed in an agar block and mounted on a metal stage. A transverse slice (500-μm thick) with an attached dorsal root was cut on a DTK-1500 Microslicer (Dosaka, Kyoto, Japan) and placed on a nylon mesh in the recording chamber. The slice was perfused continuously with Krebs solution (10 ml/min) equilibrated with a 95 % O_2_ and 5 % CO_2_ gas mixture at 36°C. The Krebs solution contained (in mM): NaCl, 117; KCl, 3.6; CaCl_2_, 2.5; MgCl_2_, 1.2; NaH_2_PO_4_, 1.2; NaHCO_3_, 25; and D-glucose, 11.5. Whole-cell patch-clamp recordings were made from lamina II neurons in voltage-clamp mode using patch pipette electrodes having a resistance of 10 MΩ. The patch pipette solution contained (in mM): Cs-sulfate, 110; CaCl_2_, 0.5; MgCl_2_, 2; EGTA, 5; HEPES, 5; tetraethylammonium (TEA), 5; and ATP-Mg salt, 5. Signals were amplified using an Axopatch 200B amplifier (Molecular Devices, Union City, CA) and were low-pass filtered with a 2-kHz cutoff and digitized at 5 kHz. Data were collected and analyzed using pClamp 10.0 software (Molecular Devices). All experiments were performed in voltage-clamp mode at a holding potential of −40 mV for recording exogenous NMDA current and −70 mV for recording exogenous AMPA current. To test the synaptic response, NMDA receptor-mediated excitatory postsynaptic currents (EPSCs) were observed by dorsal root electrical stimulation at +40 mV in the presence of an AMPA/kainate receptor antagonist (6-cyano-7-nitroquinoxaline-2, 3-dione, CNQX; 10 μM), a GABA_A_ receptor antagonist (bicuculline; 20 μM), and a glycine receptor antagonist (strychnine; 1 μM). AMPA receptor-mediated EPSCs were observed by dorsal root stimulation at −70 mV. Evoked EPSCs that displayed a constant latency and lack of failures with high frequency stimulation (20 Hz) were classified as monosynaptic. Drugs were applied by superfusion without alteration of the perfusion rate and temperature. NMDA (100 μM) and AMPA (10 μM) were applied to slices for 30 s, and each antidepressant was applied to slices for 3 min. Peak NMDA and AMPA currents were measured before and after each treatment and expressed as (posttreatment/pretreatment) × 100 (as percentages).

### **Immunohistochemistry**

The spinal cord slices (700 μm) from male rats (150–250 g) were perfused with Krebs solution for at least 3 h before application of drugs. Milnacipran (100 μM) was applied for 10 min before NMDA stimulation (100 μM for 5 min) and was present during NMDA stimulation. After drug treatment, the slices were fixed in 4 % paraformaldehyde for 60 min, replaced with sucrose overnight, cut in a cryostat at a thickness of 16 μm, and mounted on slide glass. Phosphorylated extracellular signal-regulated kinase 1/2 (pERK1/2) was visualized by indirect immunohistochemistry [18]. Sections were incubated with rabbit anti-pERK1/2 antibody (Cell Signaling Technology, Danvers, MA; 1:1000) for 2 days at 4 °C. The sections were incubated with biotinylated anti-rabbit secondary antibody (Vector Laboratories, Burlingame, CA; 1:400) for 4 h at room temperature. Signals were visualized with Vectastain ABC systems (Vector Laboratories) following the manufacturer’s instruction.

Signals were analyzed under a microscope-digital camera system (Nikon, Tokyo, Japan). Experimenters who were unaware of the experimental protocol counted cells in a blinded manner. At least five nonadjacent sections were randomly selected for cell count. The number of pERK1/2-positive neurons in the superficial dorsal horn from each of five sections was averaged for each animal.

### **Preparation of Drugs**

The drugs used in this study were milnacipran, citalopram, desipramine (provided by Asahi Kasei Corporation, Osaka, Japan), NMDA, AMPA, CNQX, bicuculline, strychnine, WAY100635 maleate salt (Sigma-Aldrich, St. Louis, MO) and tetrodotoxin (TTX), yohimbine hydrochloride (Wako, Osaka, Japan). Citalopram and bicuculline were first dissolved in dimethyl sulphoxide (DMSO) at 1000 times the concentrations to be used. The other drugs were first dissolved in distilled water at 1000 times the concentrations to be used, and then these drugs were diluted to the final concentration in Krebs solution immediately before use.

### **Statistical Analysis**

Data are expressed as means ± SEM. Statistical significance was determined as *P* < 0.05 using either the Student’s paired *t*-test or a two-way analysis of variance (ANOVA), followed by a Student-Newman-Keuls post hoc test for multiple comparisons.

## **Results**

### **Milnacipran Suppresses NMDA-Induced Thermal Hyperalgesia**

**Figure 1:**
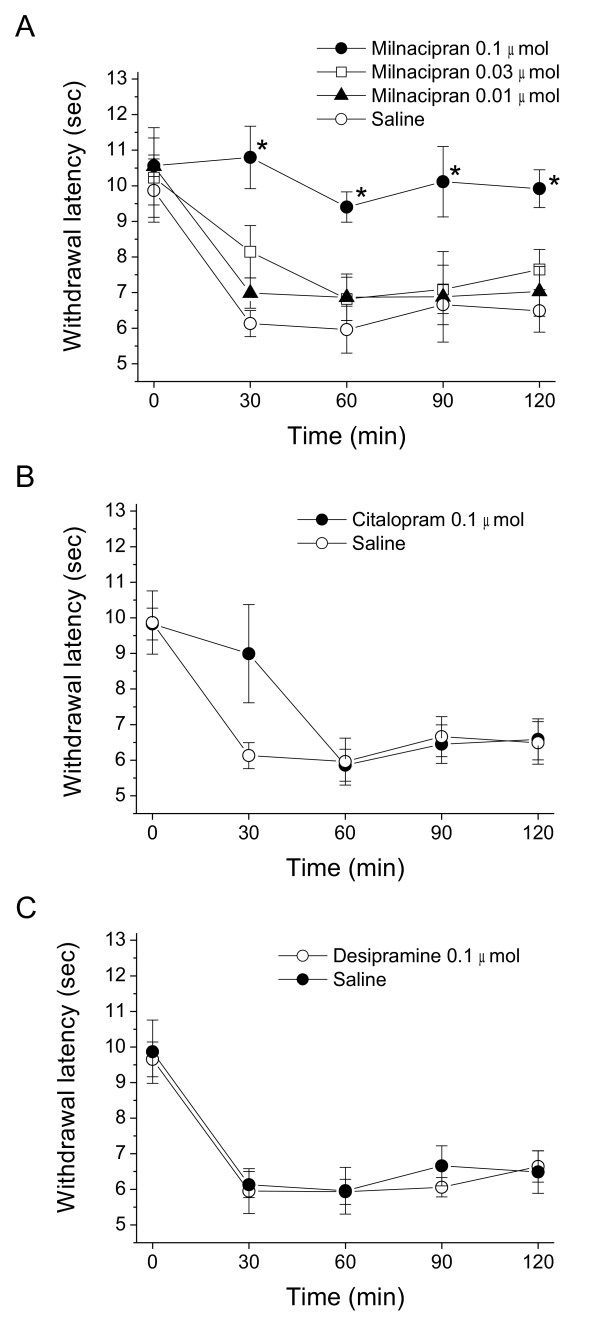
**Milnacipran suppresses NMDA-induced thermal hyperalgesia (B)or desipramine + NMDA(C)on the paw withdrawal latency against thermal nociceptive stimuli.** Thermal hyperalgesia was induced by intrathecal injection of NMDA. Saline or each drug was injected 15 min before NMDA. Paw withdrawal latencies are expressed as mean ± SEM for six rats in each group. * *P* < 0.05 compared with saline-treated group at each time point by a Student-Newman-Keuls post hoc after two-way ANOVA.

Intrathecal injection of saline or each antidepressant alone did not alter PWL (data not shown). Intrathecal injection of 1 μg NMDA 30 min after saline injection produced thermal hyperalgesia 30 min after the NMDA injection compared with the pre-value (9.87 ± 0.89 to 6.13 ± 0.89 s, *P* < 0.01). Mechanical allodynia was not induced by intrathecal injection of NMDA. Although the hyperalgesia induced by intrathecal administration of NMDA is a rapid and transient in a tail-flick test [19], the reduction in PWL continued up to 120 min after NMDA injection in the present study (saline group, Figure 1). Intrathecal injection of milnacipran followed by intrathecal injection of NMDA suppressed thermal hyperalgesia in a dose-dependent manner at doses from 0.01 to 0.1 μmol (*P* < 0.05 by two-way ANOVA, Figure. 1A). In contrast, citalopram (0.1 μmol) did not inhibit thermal hyperalgesia during the testing period (*P* = 0.25 by two-way ANOVA; Figure 1B). Similarly, desipramine (0.1 μmol) did not suppress NMDA-induced thermal hyperalgesia (*P* = 0.62 by two-way ANOVA, Figure 1C).

### **Milnacipran Inhibits NMDA, but not AMPA Receptor-Mediated Responses in Dorsal Horn Neurons**

**Figure 2:**
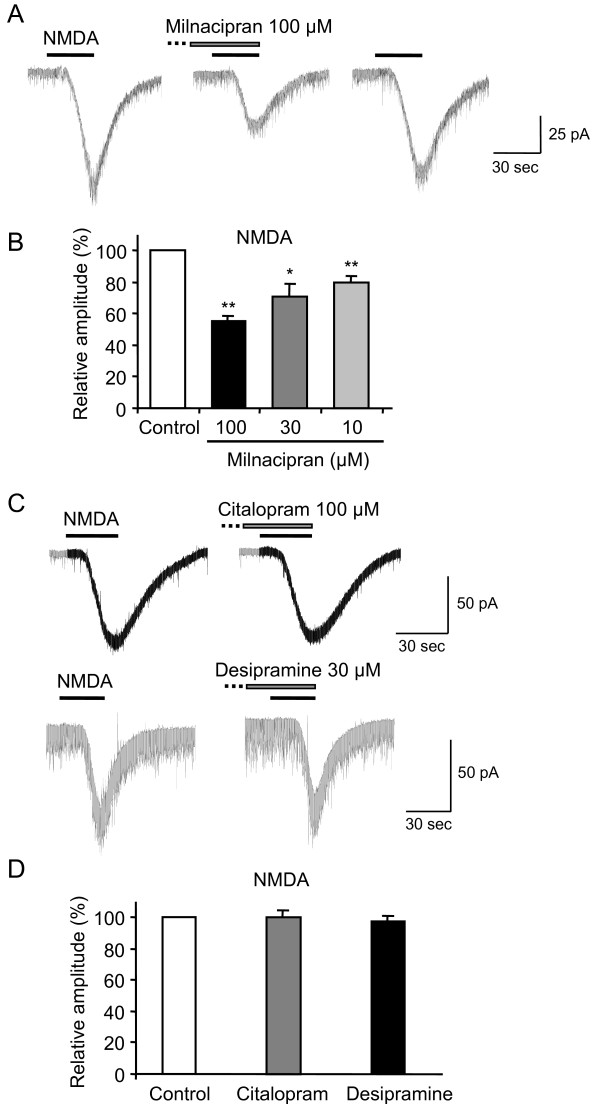
**(A) Milnacipran inhibits the response to exogenous NMDA.** Milnacipran reversibly inhibited NMDA-induced currents. In this figure and subsequent figures, the horizontal bars above the chart recordings indicate the duration of drug superfusion. **(B)** The relative amplitudes were shown in the presence of milnacipran. ** *P* < 0.01, * *P* < 0.05 **(C)** Neither citalopram nor desipramine affected the amplitudes of NMDA-induced currents. **(D)** Comparison of the NMDA-induced currents in control and in the presence of citalopram or desipramine.

**Figure 3:**
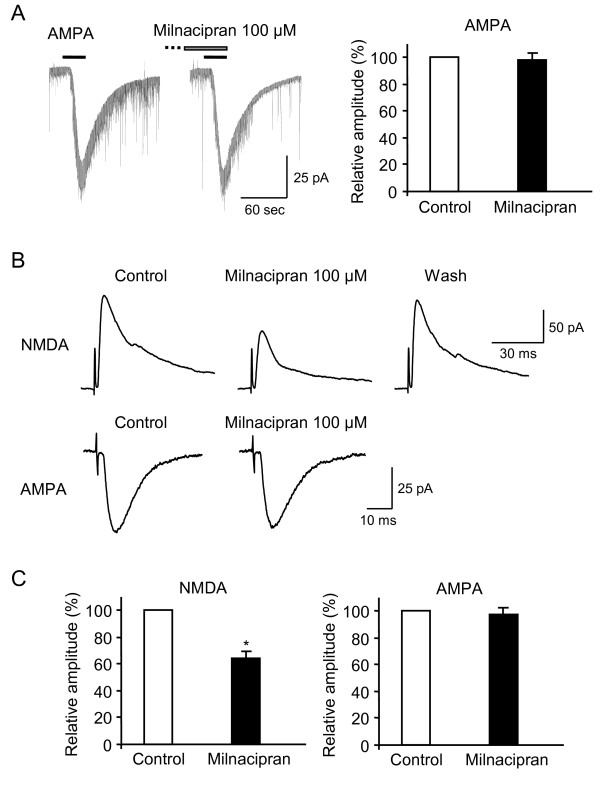
**(A) Milnacipran does not inhibit AMPA receptor-mediated responses in dorsal horn neurons.** Milnacipran did not inhibit AMPA-induced currents. **(B)** Representative traces of dorsal root stimulation evoked monosynaptic NMDA- and AMPA-mediated EPSCs. NMDA-mediated EPSCs were recorded at +40 mV. AMPA-mediated EPSCs were recorded at −70 mV. Milnacipran reversibly decreased the amplitudes of NMDA-, but not AMPA-mediated EPSCs. **(C)** Comparison of the NMDA- and AMPA-mediated EPSCs in control and in the presence of milnacipran. * *P* < 0.01.

To study the effects of milnacipran on excitatory synaptic transmission, whole-cell patch-clamp recordings were made from rat lamina II neurons. Milnacipran did not alter the level of holding current required to maintain neurons at −40 mV and −70 mV, respectively. Milnacipran had no effect on the amplitude (102 ± 5 % of control, n = 4, *P =* 0.81) and frequency (97 ± 6 % of control, n = 4, *P =* 0.62) of spontaneous EPSCs at −70 mV. Exogenous application of NMDA (100 μM, 30 s, at −40 mV) elicited an inward current in neurons (Figure 2A, C), reflecting the activation of NMDA receptors. To confirm that NMDA-induced currents were postsynaptic phenomena, we examined the currents in the presence of TTX to remove any possible influence of the NMDA receptors on presynaptic neurons. Since TTX (0.5 μM) did not affect the amplitudes of NMDA-induced currents (103 ± 4 % of control, n = 5; *P* = 0.84; data not shown), the currents were exclusively postsynaptic. Therefore, the following experiments were done in the absence of TTX. Pre-application of milnacipran (100 μM) for 3 min reduced the amplitudes of NMDA-induced currents to 56 ± 3 % (n = 10, *P* < 0.01; Fig. 2A, B) of the control values. These effects of milnacipran were reversible, and the amplitudes of currents recovered to the control values within 5–10 min (Figure 2A). At a lower concentration of 10 or 30 μM, milnacipran also decreased NMDA-induced currents (80 ± 4 % of the control, n = 5, *P* < 0.01; 71 ± 8 % of the control, n = 6, *P* < 0.05, respectively; Figure 2B). To elucidate whether the observed effects were specific for milnacipran, we examined other antidepressants; i.e., citalopram and desipramine. Pre-application of citalopram (100 μM) for 3 min failed to reduce the amplitudes of NMDA-induced currents (100 ± 5 % of control, n = 5, *P =* 0.96; Figure 2C, D). Pre-application of desipramine (30 μM) for 3 min also failed to reduce the amplitudes of NMDA-induced currents (98 ± 4 % of control, n = 5, *P =* 0.57; Figure 2C, D). In contrast, milnacipran (100 μM) had no effect on the amplitudes of exogenously applied AMPA (10 μM, 30 s, at −70 mV)-induced currents (98 ± 5 % of control, n = 7, *P =* 0.63; Figure 3A). These results suggest that milnacipran, but not the other antidepressants, inhibited NMDA, but not AMPA receptor-mediated responses in lamina II neurons.

We next tested the effects of milnacipran on the amplitudes of dorsal root stimulation evoked EPSCs. Milnacipran (100 μM) inhibited the amplitudes of monosynaptic NMDA-mediated EPSCs to 64 ± 5 % (n = 4, *P* < 0.01; Figure 3B, C) of the control values. In contrast, milnacipran (100 μM) did not inhibit the amplitudes of monosynaptic AMPA receptor-mediated EPSCs (97 ± 5 % of control, n = 5, *P =* 0.62; Figure 3B, C). To exclude the possibility that milnacipran inhibited the amplitudes of NMDA-mediated EPSCs as a result of the blockade of 5-HT and NA reuptake, we examined the NMDA-mediated EPSCs in the presence of 5-HT_1A_ receptor antagonist, WAY100635 (10 μM), and α2 receptor antagonist, yohimbine (1 μM). The concentrations of WAY100635 and yohimbine at the concentrations used here are sufficiently high to block the 5-HT_1A_[20] and α2 receptors [21], respectively. However, milnacipran also inhibited the amplitudes of NMDA-mediated EPSCs to 69 ± 8 % (n = 3, *P* < 0.01) of the control values.

### **Effects of Antidepressants on ERK Activation in the Spinal Cord**

**Figure 4:**
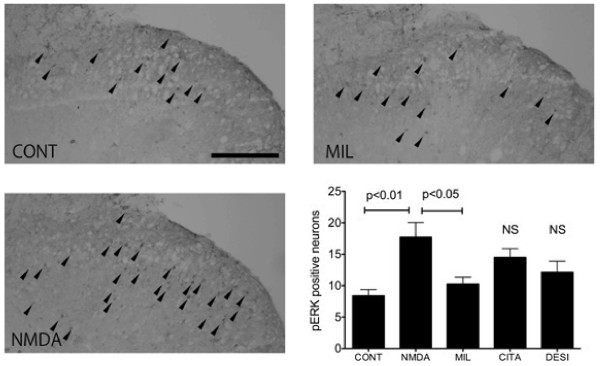
**Effects of antidepressants on ERK activation in the spinal cord.** ERK activation was significantly suppressed by simultaneous treatment with milnacipran compared with the NMDA-alone group, but not with citalopram or desipramine. Arrow indicates the pERK-positive neurons. NS = not significant.

The slice preparation offers a reliable condition under which to study pERK expression [22,23]. In addition, multiple slices can be prepared from each spinal cord segment, and milnacipran can be applied in a known condition *in vitro*. The slices were perfused for more than 3 h before stimulation to reduce possible pERK background caused by slice preparation [22,23]. There were very few pERK-positive neurons noted in control spinal cord slices (Figure 4).

In the present research, pERK-positive neurons were occasionally observed in the control dorsal horn. Bath application of NMDA (100 μM) for 5 min produced activation of pERK in the superficial dorsal horn neurons. Distribution of NMDA-evoked pERK expression was similar to that of previous observations [22]. The number of pERK-positive neurons significantly increased in the NMDA-treated group (*P* < 0.01 vs. control, Figure 4). The number of pERK-positive neurons in the group treated with milnacipran (100 μM), but not with citalopram (100 μM) or desipramine (30 μM) followed by NMDA was significantly lower compared with the NMDA-alone group (*P* < 0.05, Figure 4).

## Discussion

We demonstrated that intrathecal administration of milnacipran, but not citalopram or desipramine mediated an inhibition of NMDA-induced thermal hyperalgesia. Moreover, we documented the inhibition of NMDA-mediated currents by milnacipran, but not by citalopram or desipramine in spinal lamina II. We also demonstrated that activation of pERK induced by NMDA was significantly suppressed by milnacipran in dorsal horn neurons. Taken together, these findings indicate that milnacipran has a direct antinociceptive effect in the spinal cord through its modulation of NMDA receptors.

Some investigators have reported interactions between TCAs and NMDA receptors in nociceptive transmission. Eisenach and Gebhart [5] reported that intrathecal administration of amitriptyline reversed thermal hyperalgesia via NMDA receptor antagonism in a rat model of inflammation. Kawamata et al. [24] also using the rat model of inflammation, reported that intrathecally injected desipramine produced analgesia unrelated to NA reuptake inhibition. These reports suggest that TCAs exert a direct inhibitory effect on NMDA receptors to produce analgesia in the spinal cord. TCAs may not only inhibit 5-HT and NA reuptake, but could also interact with various receptors. It has been shown that TCAs block sodium channels [25,26] as well as voltage-dependent calcium channels [27,28] and that TCAs inhibit adenosine reuptake [29]. Further, most TCAs have affinity for opioid [30], NA, 5-HT, histamine, and muscarinic acetylcholine receptors [31]. Therefore, these various mechanisms of action of TCAs might contribute to antinociceptive effects in some kinds of chronic pain models.

Desipramine did not suppress NMDA-induced thermal hyperalgesia in the present study. However, Hwang and Wilcox [6] reported that desipramine was antinociceptive in three nociceptive tests, tail-flick test, intrathecal substance P-induced behavioral test and intradermal hypertonic saline-induced behavioral test. Moreover, they indicated that the analgesic effect by desipramine probably involves blockade of monoamine reuptake. This discrepancy between our result and that of previous study is likely to be due to different underlying mechanisms of desipramine.

Although there is some evidence that TCAs block NMDA receptor-mediated responses, the site of action is controversial. Based on radioligand binding studies, Reynolds and Miller [32] have suggested that TCAs act at the Zn^2+^ recognition site on the NMDA receptor. Sills and Loo [33] have reported that TCAs bind with higher affinity to the phencyclidine binding site on the NMDA receptor. Moreover, Sernagor et al. [34] have reported that desipramine blocked NMDA-induced currents in hippocampal neurons by acting on the open channel. In contrast to TCAs, milnacipran has no relevant affinity for any other receptors, including α-adrenergic, 5-HT, histamine, muscarinic acetylcholine, opioid, or NMDA receptors [35]. However, Shuto et al. [36] reported that milnacipran (IC50 = 6.3 μM), is a class of noncompetitive NMDA receptor antagonist, although the binding affinity of milnacipran for the NMDA receptor is not strong. Although there is no evidence that milnacipran binds to the NMDA receptor, it is assumed that it may act at the recognition site for Zn^2+^ or at the phencyclidine binding site of the NMDA receptor. Further study is required to clarify this point.

Milnacipran inhibits the presynaptic reuptake of monoamines, 5-HT and NA with an IC50 of 100 to 200 nM, respectively in the brain [37]. However, in this study, milnacipran has the antagonistic effect on NMDA-mediated responses in the spinal cord at a concentration of 10–100 μM. Some previous studies [32,36,38] indicate that TCAs also inhibits the NMDA receptors in the brain at a concentration of 10–100 μM. Therefore, it is likely that the concentration of inhibiting the NMDA receptors by milnacipran is higher than that of inhibiting reuptake of the monoamines.

In the present study, milnacipran reduced the amplitudes of exogenously applied NMDA-induced currents in lamina II neurons. Moreover, milnacipran inhibited the amplitudes of dorsal root stimulation evoked NMDA-mediated EPSCs. There are no differences in the degree of depression by milnacipran between NMDA induced-current and dorsal root stimulation evoked NMDA-mediated EPSCs. These results suggest that synaptic and extra synaptic NMDA receptors in dorsal horn neurons are similarly modulated by milnacipran. Moreover, milnacipran inhibited the amplitudes of NMDA-mediated EPSCs in the presence of 5-HT antagonist and α2 receptor antagonist. Therefore, it is unlikely that an antagonistic effect of milnacipran on NMDA receptors is mediated by 5-HT or NA receptors.

We observed that the highest dose of intrathecal milnacipran completely reversed thermal hyperalgesia induced by NMDA. In contrast, desipramine and citalopram did not produce any inhibitory effect, although the concentrations of desipramine and citalopram in this study are sufficiently high to inhibit reuptake of 5-HT or NA, respectively. These results suggest that increases in 5-HT or NA alone in the spinal cord have no effect on NMDA-induced thermal hyperalgesia. The antagonistic action of milnacipran for NMDA receptors may produce additional effects for some types of chronic pain. Previous studies demonstrated that intrathecal administration of milnacipran produced antiallodynic effects in rats with peripheral nerve injury [13,39,40], and that the effect was not completely reversed by an α2 receptor antagonist or a 5-HT receptor antagonist [13]. Therefore, it is conceivable that an antagonistic action for NMDA receptors contributes to the antiallodynic effect of milnacipran. Further studies are required to clarify the molecular mechanisms underlying the inhibitory effect of milnacipran on NMDA-mediated responses in dorsal horn neurons. In addition, to elucidate whether the observed effects were specific for milnacipran, further investigations using another SNRI are necessary to resolve this question.

ERK activation is detected in the spinal dorsal horn neurons after stimulation of nociceptive primary afferents and contributes to the development of central sensitization [22]. Activation of the NMDA receptor is partly involved in ERK induction following nociceptive stimulation [22]. Analgesic drugs such as local anesthetics [41], opioids, or cannabinoids [42] inhibit ERK induction in the spinal cord. In the present study, we demonstrated that milnacipran inhibited ERK induction following application of NMDA in the spinal cord. This result is consistent with our behavioral data showing that milnacipran attenuated thermal hyperalgesia following intrathecal NMDA injection. Our electrophysiological data clearly indicate that the direct inhibitory effect of milnacipran on NMDA-mediated current underlies these phenomena.

We show in the present study that milnacipran has an antagonistic effect of NMDA receptors in the spinal cord when administered intrathecally. However, it is not clear whether milnacipran has the similar effect in other central and peripheral tissue such as brain and skin. Moreover, NMDA receptor antagonists such as ketamine and phencyclidine have psychotomimetic or anti-depressant properties in humans when administered systemically. There have been no studies demonstrating that milnacipran has these similar properties in humans. Therefore, further study is required to clarify this point.

NMDA glutamate receptors are one of the major receptor channel types mediating rapid excitatory neurotransmission in the central nervous system. This receptor is composed of subunits from at least two families, NR1 and NR2. The NR1 subunit is essential for the function of NMDA receptors and is ubiquitously expressed in most neurons. The functional properties of NMDA receptors are determined by the NR2 subunit composition (NR2A–2D). Previous reports have demonstrated that desipramine inhibited NMDA-evoked responses in hippocampal neurons [32,43], but not in the present study. The respective NR2 subunits show different expression patterns in various regions of the brain and spinal cord. Whereas NR2A and NR2B subunits are prominent in the hippocampus [44], these subunits are not identified in spinal dorsal horn neurons [45]. This different composition of NMDA receptors may underlie the variability among tissues in the effects of desipramine on NMDA receptor-responses.

NMDA glutamate receptors also play a key role in central sensitization in chronic pain [3,4]. The pursuit of an NMDA receptor antagonist for the relief of chronic pain dates from the late 1980s when it was shown that NMDA antagonists inhibit the “wind-up” response [46,47]. The central sensitization that occurs in the spinal dorsal horn is held to be an important event in the pathway leading to neuropathic pain. For this reason, ketamine is currently widely applied in the treatment of neuropathic pain and for some kinds of chronic pain, including fibromyalgia [48]. Although there is little evidence that NMDA receptor antagonism is involved in the antinociceptive effect of milnacipran, milnacipran has been widely used in patients with fibromyalgia and has provided them with pain relief [49,50]. These effects of milnacipran for chronic pain have been previously discussed from the perspective of one aspect: balanced inhibition of the reuptake of 5-HT and NA. However, in the present study, our data suggest that milnacipran not only inhibits 5-HT and NA reuptake, but also acts as an NMDA receptor antagonist. Milnacipran may have the potential to prevent or suppress chronic pain related to central sensitization, especially when injected into intrathecal or epidural space.

## Conclusions

In conclusion, the present study suggests that milnacipran, but not citalopram and desipramine, inhibits NMDA-induced glutamatergic transmission in rat dorsal horn neurons. These results suggest that not only is inhibition of 5-HT and NA reuptake an important factor for the analgesia induced by milnacipran in the spinal cord, but that inhibition of glutamate NMDA receptors is critical as well.

## Competing interests

The authors declare that they have no competing interests.

## Authors' contributions

MK and HO performed the animal surgery and behavioral test. TK carried out *in vitro* patch-clamp recordings. MS and FA conducted immunohistochemistry. SS performed the statistical analysis. TK, HO and FA drafted the manuscript. All authors read and approved the final manuscript.
